# Organizational dehumanization and gig workers’ outcomes: a moderated mediation model of psychological distress and family recognition of work

**DOI:** 10.3389/fpsyg.2026.1851798

**Published:** 2026-08-05

**Authors:** Hu Ying, Lidong He, Jie Liu, Xiaofu Pan

**Affiliations:** 1 College of State Governance, Southwest University, Chongqing, China; 2 School of Marxism Studies, Chongqing University of Chinese Medicine, Chongqing, China; 3 Department of Psychology, Wuhan University, Wuhan, China

**Keywords:** commitment to service quality, family recognition of work, flourishing, gig worker, job demands-resources model, organizational dehumanization, psychological distress

## Abstract

**Introduction:**

Platform work is increasingly organized through algorithmic rules and standardized performance systems that may leave workers feeling treated as replaceable instruments rather than as full persons. Drawing on the Job Demands-Resources (JD-R) model, this study examined whether psychological distress mediates the associations between organizational dehumanization and gig workers’ flourishing and commitment to service quality, and whether family recognition of work moderates this process.

**Methods:**

Data were collected from married food-delivery and ride-hailing platform workers in China across three waves separated by 1-week intervals. The analytic sample comprised 281 participants.

**Results:**

Organizational dehumanization was positively associated with psychological distress, which in turn was negatively associated with both flourishing and commitment to service quality. Family recognition of work weakened the association between organizational dehumanization and psychological distress and attenuated the indirect associations between organizational dehumanization and both outcomes through psychological distress.

**Discussion:**

Family recognition of work may serve as a supplementary cross-domain resource for gig workers, while reducing dehumanizing conditions in platform work remains the primary intervention target.

## 1 Introduction

Organizational dehumanization refers to employees’ perception that their organization treats them as instruments or tools rather than as full human beings (Haslam and Loughnan, 2014; Bell and Khoury, 2016). Prior research has linked organizational dehumanization to emotional labor (Nguyen et al., 2022a,b), negative work attitudes and behaviors (Nguyen and Stinglhamber, 2020, 2021), work-related psychological strain (Caesens and Stinglhamber, 2019; Lagios et al., 2022; Sarwar et al., 2021; Taskin et al., 2019), psychosomatic symptoms (Caesens et al., 2016), and emotional exhaustion (Stinglhamber et al., 2021). This body of work suggests that organizational dehumanization is a meaningful workplace stressor with implications for employees’ wellbeing and work-related functioning.

China’s platform economy has expanded rapidly in recent years, alongside a substantial increase in gig work. Although gig workers often lack stable organizational membership, their work remains shaped by platform rules, algorithmic allocation, performance ratings, and standardized service requirements. Unlike traditional employees, gig workers may experience organizational control without stable organizational membership, making dehumanization both less visible and more difficult to contest. These features may make organizational dehumanization particularly salient in platform work. Workers may feel that platforms treat them as replaceable labor units or data points rather than as workers with dignity and voice. Platform work therefore provides a useful context for examining how organizational dehumanization relates to workers’ psychological wellbeing and service-related outcomes.

Recent research on freelancers situates gig and platform-based work within a broader shift toward non-traditional employment arrangements, including independent contracting, project-based work, and other forms of work outside standard employment relationships. This literature has examined how human resource practices can support freelancer success (Rabenu et al., 2026), how managers perceive and collaborate with freelancers (Zadik et al., 2019), how freelancers’ psychological contracts are shaped within employment relationships (van den Groenendaal et al., 2023), and how the characteristics of “gigs” influence freelancers’ needs and work experiences (Barlage et al., 2023). Collectively, these studies suggest that workers outside standard employment may benefit from flexibility and autonomy, while also facing limited organizational support, weak organizational integration, fragmented work relationships, and dependence on clients or platform-mediated practices. Although food-delivery and ride-hailing platform workers differ from highly skilled professional freelancers, both groups reflect forms of work in which individuals remain connected to organizations or platforms while lacking many of the protections, resources, and recognition associated with standard employment. This perspective helps position the present study within broader debates on organizational control, recognition, and marginalization in nonstandard work.

The Job Demands-Resources (JD-R) model defines job demands as physical, psychological, social, and organizational aspects of work that require sustained effort and are associated with psychological or physiological costs (Bakker and Demerouti, 2007, 2017). From this perspective, organizational dehumanization can be viewed as a relational job demand because it threatens workers’ dignity, agency, and sense of being valued as human beings. This demand is relational because it concerns how workers are recognized, valued, and granted agency, rather than only the amount or difficulty of work. For gig workers, this demand may be intensified by algorithmic management and limited access to organizational support. Repeated experiences of being treated as a tool or replaceable unit may consume psychological resources and contribute to psychological distress.

Psychological distress is unlikely to fully account for the associations between organizational dehumanization and worker outcomes. Perceiving oneself as a tool or replaceable unit may also undermine occupational dignity and weaken workers’ perceived reciprocal obligation toward the platform and its service goals. Accordingly, we expected psychological distress to partially, rather than fully, mediate the associations of organizational dehumanization with flourishing and commitment to service quality. This expectation also provides a theoretical basis for direct associations to remain after psychological distress is considered.

Gig workers may have limited access to organizational resources that are more readily available to traditional employees, such as supervisor support, team belonging, and formal organizational support. Under such conditions, resources outside the workplace may become especially relevant. The Work-Home Resources Model proposes that resources generated in one life domain can help protect or replenish resources in another domain (Ten Brummelhuis and Bakker, 2012). From this perspective, the family may serve as an important nonwork source of support for gig workers. This argument is also consistent with the stress-buffering perspective of social support, which suggests that social support can reduce the psychological costs of stressful experiences (Cohen and Wills, 1985).

Given the strong family orientation in many Chinese workers’ lives, family members’ understanding, respect, and validation may be especially important for Chinese gig workers. Family recognition of work refers to the extent to which family members recognize and value one’s work. Unlike general family support, family recognition of work specifically validates the social worth and dignity of the worker’s occupation. When gig workers receive such recognition, they may be more likely to view their work as meaningful and worthy of respect. This recognition may help maintain occupational dignity and self-worth when workers experience dehumanizing treatment from platforms. Thus, family recognition of work may serve as a resource across work and family domains that buffers the association between organizational dehumanization and psychological distress. Specifically, we expected the positive association between organizational dehumanization and psychological distress to be weaker when family recognition of work is higher. We further expected family recognition of work to attenuate the indirect associations of organizational dehumanization with flourishing and commitment to service quality through psychological distress.

**FIGURE 1:**
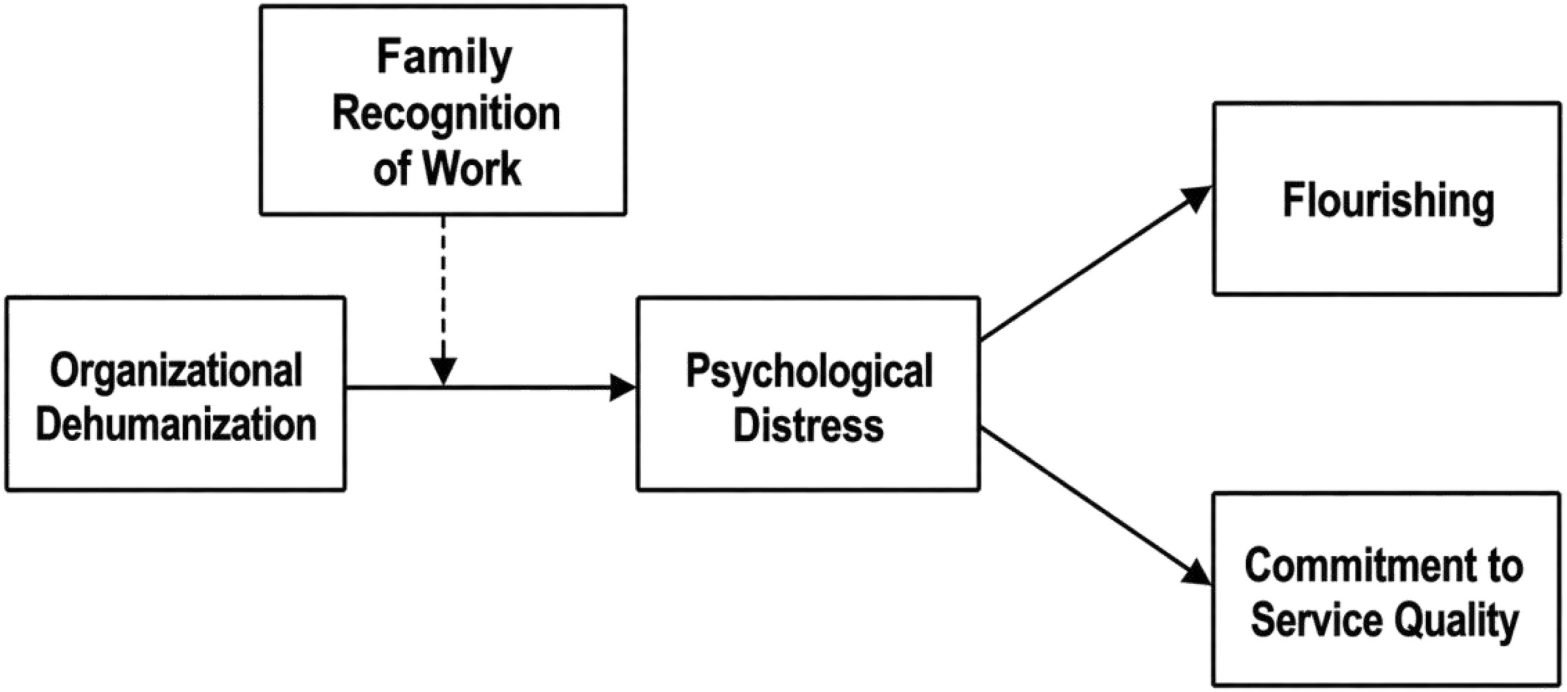
Theoretical model.

The present study examines this moderated mediation model among Chinese gig workers using a three-wave design (see Figure 1). We propose that organizational dehumanization is positively associated with psychological distress, which in turn is negatively associated with flourishing and commitment to service quality. We further propose that family recognition of work moderates the association between organizational dehumanization and psychological distress, thereby weakening the indirect associations between organizational dehumanization and the two outcomes. The study contributes to the literature by extending organizational dehumanization research to platform-based gig work, conceptualizing organizational dehumanization as a relational job demand within the JD-R framework, and examining family recognition of work as a resource across work and family domains that may buffer, but not eliminate, the psychological costs associated with organizational dehumanization.

*H1*: Organizational dehumanization is positively associated with psychological distress.

*H2a*: Psychological distress is negatively associated with flourishing.

*H2b*: Psychological distress is negatively associated with commitment to service quality.

H3a: Psychological distress partially mediates the association between organizational dehumanization and flourishing.

*H3b*: Psychological distress partially mediates the association between organizational dehumanization and commitment to service quality.

*H4*: Family recognition of work moderates the association between organizational dehumanization and psychological distress, such that the association is weaker when family recognition is higher.

*H5a*: The indirect association between organizational dehumanization and flourishing through psychological distress is weaker when family recognition is higher.

*H5b*: The indirect association between organizational dehumanization and commitment to service quality through psychological distress is weaker when family recognition of work is higher.

## 2 Materials and methods

### 2.1 Participants and procedure

Participants were recruited through Credamo, a Chinese online survey platform similar to Turk, and were remunerated through the platform. The study targeted married gig workers in the food-delivery and ride-hailing industries. We focused on married workers because family recognition of work was a central construct in the study and was expected to be especially salient for workers embedded in ongoing family roles and responsibilities. During recruitment, eligibility was restricted to participants who were currently working as platform workers in either food-delivery or ride-hailing services and who reported being married.

To verify that participants were actual platform workers, they were required to report their platform work tenure, daily online working time on the platform, and current platform rating. These work-related indicators were used to confirm participants’ platform-work experience and to screen out responses inconsistent with actual platform employment. In addition, invalid responses were excluded if participants failed verification items, showed abnormal response times, gave patterned or repetitive responses, failed to report required platform-work information, provided duplicate participant IDs, or could not be matched across waves.

Data were collected at three time points with a 1-week interval between adjacent waves. The 1-week interval was used to reduce common method bias through temporal separation while retaining a time frame suitable for capturing short-term psychological responses to dehumanizing platform experiences. Psychological distress, flourishing, and commitment to service quality can reflect proximal psychological and attitudinal reactions to recent work experiences rather than only stable long-term tendencies. Given the frequent and immediate customer-platform interactions in food-delivery and ride-hailing work, a 1-week interval is appropriate for capturing short-term changes or recent manifestations of these outcomes. At Time 1, participants completed measures of organizational dehumanization, family recognition of work, customer mistreatment, and demographic and work-related variables. At Time 2, participants completed the measure of psychological distress. At Time 3, participants completed measures of flourishing and commitment to service quality. Responses across the three waves were matched using participant IDs.

After data screening, 396 valid questionnaires were retained at Time 1, 341 at Time 2, and 291 at Time 3. The retention rate was 86.1% from Time 1 to Time 2 and 73.5% from Time 1 to Time 3. Attrition analyses were conducted to compare participants retained in the final sample (*n* = 291) with those who dropped out before Time 3 (*n* = 105) on available Time 1 variables. The two groups did not differ significantly in age, education, platform work tenure, daily online working time, customer mistreatment, or family recognition of work, *ps* > 0.05. However, participants retained in the final sample reported lower Time 1 organizational dehumanization (*M* = 3.67, *SD* = 1.59) than those who dropped out before Time 3 (*M* = 4.06, *SD* = 1.56), *t*(187.90) = −2.18, *p* = 0.030, Cohen’s *d* = −0.25. This result suggests that attrition was not completely random, as participants who perceived higher organizational dehumanization were more likely to drop out before Time 3. Because participants who reported higher levels of organizational dehumanization were more likely to drop out, the observed associations involving organizational dehumanization may be somewhat conservative, as selective attrition could have attenuated the estimated effects.

The final sample consisted of 291 married gig workers. Among them, 200 participants were male (68.7%) and 91 were female (31.3%). The average age was 32.57 years (*SD* = 6.78). Participants had an average platform work tenure of 3.64 years (*SD* = 2.31), and an average daily online working time of 8.4 h (*SD* = 2.38). Although the final matched sample included 291 participants, 10 participants did not report the date when they started working on the platform; therefore, platform work tenure could not be calculated for these cases. After excluding cases with missing values on the control variables, the PROCESS analyses were based on an analytic sample of *N* = 281. The study was approved by the Institutional Review Board of the Faculty of Psychology at Southwest University (Approval No.: H25080).

### 2.2 Measures

Except for psychological distress, all the measures were developed in English. Accordingly, we used a double-blind back-translation procedure (Brislin, 1973).

#### 2.2.1 Organizational dehumanization

We adopted the 11-item scale developed by Caesens et al. (2016). Because the present study focused on platform-based gig workers, participants were instructed to evaluate the main platform through which they obtained gig work. Items referring to “organization” were adapted to the platform-work context. For example, the item “My organization considers me as a tool to use for its own ends” was adapted as “My platform considers me as a tool to use for its own ends.” During the translation and adaptation process, we reviewed the items for conceptual relevance to platform work. The items were retained because they capture the core perception of being treated as an instrument or tool, which can apply to platform-based control even when workers lack traditional organizational membership. For consistency, we use the term “organizational dehumanization” throughout the manuscript, while noting that the measure was adapted to refer to workers’ main platform in the present platform-work context. Cronbach’s alpha for this scale was 0.96.

#### 2.2.2 Customer mistreatment

Customer mistreatment was measured using the scale developed by Yue et al. (2017). A sample item is “During today’s work, how many customers have got angry at you even over minor matters?” Cronbach’s alpha for this scale was 0.83.

#### 2.2.3 Psychological distress

We adopted K6 developed by Kessler et al. (2002) to measure distress. To align the measure with the 1-week interval of the study design, participants were instructed to report how often they had experienced each symptom during the past week. A sample item is “During the past week, how often did you feel that everything was an effort?” Cronbach’s alpha was 0.84.

#### 2.2.4 Family recognition of work

We adopted the 5-item scale developed by Hwang et al. (2025). A sample item is “My family recognizes the value of my work.” Cronbach’s alpha for this scale was 0.86.

#### 2.2.5 Flourishing

We adopted the 8-item scale developed by Tang et al. (2016). A sample item is “I actively contribute to the happiness and wellbeing of others.” Cronbach’s alpha for this scale was 0.89.

#### 2.2.6 Commitment to service quality

We adopted the 6-item scale developed by Lu et al. (2024). A sample item is “I will try my best to help customers.” Cronbach’s alpha for this scale was 0.78.

#### 2.2.7 Control variables

Customer mistreatment was included as a control variable because prior research has shown that customer mistreatment is associated with negative employee emotions and work outcomes (Ho and Gupta, 2014; Rupp et al., 2008). In addition, age, gender, education, platform work tenure, daily online working time were included as demographic and work-related control variables. Thus, the main PROCESS analyses controlled for age, gender, education, platform work tenure, daily online working time, and customer mistreatment.

## 3 Results

### 3.1 Confirmatory factor analysis

To examine discriminant validity, we conducted confirmatory factor analyses using item-level data from the final matched sample. The hypothesized six-factor model specified organizational dehumanization, family recognition of work, customer mistreatment, psychological distress, commitment to service quality, and flourishing as distinct latent factors. As shown in Table 1, the six-factor model showed acceptable fit, χ^2^(887) = 1233.21, χ^2^/df = 1.39, CFI = 0.945, TLI = 0.942, RMSEA = 0.037, SRMR = 0.048, and fit better than the alternative models. These results supported the discriminant validity of the focal constructs. The poor fit of the one-factor model suggested that common method bias was unlikely to fully account for the observed relationships. Nevertheless, because all variables were measured using self-report questionnaires, common method bias cannot be completely ruled out.

**TABLE 1:** Results of confirmatory factor analyses.

Model	χ ^2^	df	χ ^2^/df	CFI	TLI	RMSEA	SRMR
Six-factor model	1233.21	887	1.39	0.945	0.942	0.037	0.048
Five-factor model: Organizational dehumanization and psychological distress combined	1618.21	892	1.81	0.885	0.878	0.053	0.072
Five-factor model: Commitment to service quality and flourishing combined	1345.55	892	1.51	0.928	0.924	0.042	0.051
One-factor model	3450.38	902	3.83	0.597	0.577	0.099	0.120

### 3.2 Descriptive statistics and correlations

Table 2 presents the descriptive statistics and correlations for the focal variables. Organizational dehumanization was positively correlated with psychological distress and negatively correlated with flourishing and commitment to service quality. Family recognition of work was negatively correlated with psychological distress and positively correlated with both flourishing and commitment to service quality.

**TABLE 2:** Descriptive statistics and correlations for variables.

Variable	*M*	*SD*	1	2	3	4
1. Commitment to service quality	5.95	0.61	0.632***	−0.473***	−0.386***	−0.274***
2. Flourishing	5.70	0.85				
3. Psychological distress	1.84	0.56	−0.349***			
4. Family recognition of work	5.88	0.86	0.373***	0.403***		
5. Organizational dehumanization	3.67	1.59	−0.398***	−0.437***	0.517***	

*N* = 291. Correlations are Pearson’s r coefficients.

***p < 0.001 (two-tailed).

### 3.3 Moderated mediation analysis

We tested the moderated mediation model using PROCESS Model 7 (Hayes, 2022). Organizational dehumanization was specified as the independent variable, psychological distress as the mediator, family recognition of work as the moderator, and flourishing and commitment to service quality as the dependent variables. Age, gender, education, platform work tenure, daily online working time, and customer mistreatment were included as control variables. To keep the regression tables focused on the hypothesized paths, coefficients for control variables are omitted from Tables 3, 4 unless directly relevant; all reported models included the full set of control variables described above. Continuous predictors were mean-centered before the interaction term was created, and significant interactions were probed using simple slope analyses following Hayes (2022). Bootstrap confidence intervals were estimated using 5,000 bootstrap samples. After excluding cases with missing values on the control variables, the PROCESS analyses were based on an analytic sample of *N* = 281.

**TABLE 3:** First-stage moderation effects on psychological distress.

Predictor	β	*SE*	*t*	*p*	95% CI
Organizational dehumanization	0.423	0.108	3.92	< 0.001	[0.211, 0.636]
Family recognition of work	0.078	0.083	0.93	0.351	[−0.086, 0.242]
Organizational dehumanization × family recognition of work	−0.054	0.018	−3.02	0.003	[−0.089, −0.019]
Customer mistreatment	0.303	0.061	5.00	< 0.001	[0.184, 0.422]

Outcome variable = psychological distress. Control variables included age, gender, education, platform work tenure, daily online working time, and customer mistreatment. *N* = 281. Full model *R*^2^ = 0.438.

**TABLE 4:** Outcome models predicting flourishing and commitment to service quality.

Predictor	Flourishing β	SE	*t*	*p*	95% CI	Commitment β	SE	*t*	*p*	95% CI
Organizational dehumanization	−0.127	0.033	−3.85	< 0.001	[−0.192, −0.062]	−0.105	0.026	−3.99	< 0.001	[−0.157, −0.053]
Psychological distress	−0.533	0.094	−5.65	< 0.001	[−0.719, −0.347]	−0.181	0.076	−2.39	0.017	[−0.330, −0.032]

All models included age, gender, education, platform work tenure, daily online working time, and customer mistreatment as control variables. Model summaries: Flourishing, *R*^2^ = 0.328, *F*(8, 272) = 16.59, *p* < 0.001; commitment to service quality, *R*^2^ = 0.216, *F*(8, 272) = 9.38, *p* < 0.001. *N* = 281.

As shown in Table 3, the first-stage model predicting psychological distress was significant, *R*^2^ = 0.438, *F*(9, 271) = 23.45, *p* < 0.001. Organizational dehumanization positively predicted psychological distress, β = 0.423, SE = 0.108, *p* < 0.001, 95% CI [0.211, 0.636], supporting H1. The interaction between organizational dehumanization and family recognition of work was also significant, β = −0.054, SE = 0.018, *p* = 0.003, 95% CI [−0.089, −0.019]. Adding the interaction term explained an additional 1.9% of the variance in psychological distress, Δ*R*^2^ = 0.019, Δ*F*(1, 271) = 9.12, *p* = 0.003. Simple slope analyses showed that the positive association between organizational dehumanization and psychological distress became weaker as family recognition increased. These results supported H4.

As shown in Table 4, psychological distress negatively predicted both flourishing and commitment to service quality, supporting H2a and H2b. Organizational dehumanization remained significantly associated with both outcomes after psychological distress and the control variables were included, suggesting partial mediation. Adding psychological distress to the outcome models explained an additional 7.9% of the variance in flourishing (Δ*R*^2^ = 0.079) and an additional 1.6% of the variance in commitment to service quality (Δ*R*^2^ = 0.016). The indirect effects were further examined using bootstrap analyses.

We further examined conditional indirect effects at low (−1 *SD*), mean, and high (+1 *SD*) levels of family recognition of work. As shown in Table 5, the indirect effect of organizational dehumanization on flourishing through psychological distress was significant at all three levels of family recognition of work, supporting H3a. The effect became weaker as family recognition of work increased. The index of moderated mediation was significant, index = 0.029, *BootSE* = 0.012, 95% Boot CI [0.010, 0.056], supporting H5a. Similarly, the indirect effect of organizational dehumanization on commitment to service quality through psychological distress was significant at all three levels of family recognition of work, supporting H3b. This effect also became weaker as family recognition of work increased. The index of moderated mediation was also significant, index = 0.010, *BootSE* = 0.005, 95% Boot CI [0.002, 0.021], supporting H5b.

**TABLE 5:** Conditional indirect effects at different levels of family recognition of work.

Outcome	Family recognition of work	Indirect effect	BootSE	95% Boot CI
Flourishing	Low (−1 *SD*)	−0.081	0.022	[−0.129, −0.042]
Flourishing	Mean	−0.056	0.016	[−0.091, −0.029]
Flourishing	High (+1 *SD*)	−0.031	0.014	[−0.062, −0.006]
Commitment to service quality	Low (−1 *SD*)	−0.028	0.012	[−0.052, −0.006]
Commitment to service quality	Mean	−0.019	0.009	[−0.037, −0.004]
Commitment to service quality	High (+1 *SD*)	−0.011	0.006	[−0.025, −0.001]
Index of moderated mediation: flourishing	−	0.029	0.012	[0.010, 0.056]
Index of moderated mediation: commitment to service quality	−	0.010	0.005	[0.002, 0.021]

Bootstrap samples = 5,000. CI = percentile bootstrap confidence interval.

Overall, organizational dehumanization was associated with lower flourishing and lower commitment to service quality through higher psychological distress. Family recognition of work moderated the first-stage association between organizational dehumanization and psychological distress, thereby attenuating the indirect effects on both outcomes. Thus, H1, H2a, H2b, H3a, H3b, H4, H5a, and H5b were supported. Notably, family recognition of work reduced, but did not eliminate, the negative process associated with organizational dehumanization.

As a supplementary robustness check, we conducted an inverse probability weighting analysis to assess whether attrition affected the main conclusions. This analysis was conducted because attrition analyses indicated that participants with higher Time 1 organizational dehumanization were less likely to remain in the final sample. The probability of inclusion in the final PROCESS analytic sample was estimated using available Time 1 variables, including organizational dehumanization, family recognition of work, customer mistreatment, gender, education, daily online working time, age, and platform work tenure. Stabilized inverse probability weights were then applied to the moderated mediation analyses. The weighted results were substantively consistent with the main analyses. The interaction between organizational dehumanization and family recognition of work remained significant, β = −0.055, *SE* = 0.019, *p* = 0.003. The indices of moderated mediation also remained significant for flourishing, index = 0.029, *BootSE* = 0.012, 95% Boot CI [0.010, 0.056], and commitment to service quality, index = 0.010, *BootSE* = 0.005, 95% Boot CI [0.002, 0.022]. These results suggest that the main conclusions were not fully driven by attrition.

## 4 Discussion

This study examined whether organizational dehumanization is associated with gig workers’ flourishing and commitment to service quality through psychological distress, and whether family recognition of work moderates this process. Drawing on the JD-R model, organizational dehumanization was conceptualized as a relational job demand and psychological distress as a proximal strain response. The results suggested that organizational dehumanization was positively associated with psychological distress, which was negatively associated with both flourishing and commitment to service quality. Family recognition of work weakened the association between organizational dehumanization and psychological distress, and the indices of moderated mediation were significant for both outcomes. Together, these findings suggest that dehumanizing platform conditions are linked not only to workers’ psychological strain but also to reduced positive functioning and service-related commitment.

The findings support psychological distress as a partial mediator between organizational dehumanization and gig workers’ outcomes. Consistent with the health impairment process of the JD-R model (Bakker and Demerouti, 2007, 2017), organizational dehumanization may function as a psychologically costly job demand. In platform work, dehumanization may be reflected in workers’ experiences of algorithmic allocation, performance ratings, standardized rules, and limited worker voice. Such experiences may threaten workers’ dignity and self-worth, thereby increasing psychological distress. Psychological distress was then associated with lower flourishing and weaker commitment to service quality. At the same time, the direct associations between organizational dehumanization and both outcomes remained significant after psychological distress was included, suggesting that psychological distress is not the only relevant mechanism.

The remaining direct associations between organizational dehumanization and both outcomes may reflect mechanisms beyond psychological distress. Organizational dehumanization may undermine workers’ occupational dignity and self-worth, reduce the perceived meaningfulness of platform work, or weaken their sense of reciprocal obligation toward platform service goals. These mechanisms were not directly measured in the present study and should be examined in future research.

The results also suggest that family recognition of work may serve as a resource across work and family domains. Gig workers often have limited access to organizational resources such as supervisor support or team belonging. In this context, recognition from family members may help workers maintain a sense of dignity and social worth because it validates the value of their work outside the platform domain. Consistent with the Work-Home Resources Model (Ten Brummelhuis and Bakker, 2012) and the stress-buffering perspective of social support (Cohen and Wills, 1985), family recognition of work weakened the link between organizational dehumanization and psychological distress. This buffering pattern should be interpreted with care. Organizational dehumanization remained positively associated with psychological distress even when family recognition of work was high. Thus, family recognition of work may reduce, but does not remove, the psychological costs associated with dehumanizing platform work.

The conditional indirect effects further showed that family recognition of work attenuated the indirect associations between organizational dehumanization and both outcomes through psychological distress. Because the present study did not directly compare the magnitude of the conditional indirect effects across outcomes, we refrain from drawing conclusions about whether the buffering process is stronger for one outcome than for the other.

These findings make several contributions. First, this study extends organizational dehumanization research beyond traditional employment by suggesting that platform workers may experience dehumanization despite lacking stable organizational membership. Second, it specifies organizational dehumanization as a relational job demand within the JD-R framework. Third, it identifies family recognition of work as a cross-domain resource that may help preserve dignity when platform work is experienced as dehumanizing. Together, these contributions also connect the present study to recent research on freelancers and nontraditional employment by suggesting that platform-mediated work may entail not only flexibility and autonomy, but also dignity-related and psychological costs when organizational control is experienced as dehumanizing.

The primary intervention target should be platform practices that produce dehumanizing experiences. Family and community recognition may provide supplementary support, but should not be treated as a substitute for platform responsibility.

Several limitations should be noted. First, although the three-wave design provided temporal separation, all variables were measured using self-report questionnaires. Moreover, the 1-week intervals capture short-term psychological responses and do not establish longer-term developmental processes. Therefore, the findings should not be interpreted as definitive evidence of causal relationships. Future research could use experimental, diary, or multi-source designs to further examine the proposed process. Second, the study included only married gig workers because family recognition of work was expected to be especially meaningful for workers embedded in family roles. This sampling decision fits the theoretical focus of the study, but the findings may not generalize to unmarried gig workers. In particular, younger or unmarried platform workers may draw more heavily on peer, community, or platform-based sources of recognition than on family recognition of work. Future research should examine whether the present moderated mediation process generalizes to these groups and whether different sources of recognition play similar buffering roles. Third, attrition analyses showed that participants with higher Time 1 organizational dehumanization were more likely to drop out before Time 3. Although the inverse probability weighting robustness check produced substantively similar results, this nonrandom attrition may still have attenuated the observed associations involving organizational dehumanization, because workers experiencing stronger dehumanization were less likely to complete all three waves. Fourth, although the organizational dehumanization scale was adapted to refer to workers’ main platform, the measure was originally developed in traditional organizational settings. Future research should develop and validate platform-specific measures of dehumanization that capture algorithmic management, platform communication, and worker voice. Fifth, although the analyses controlled for several demographic and work-related variables, other factors, such as personality, resilience, income dependence on gig work, platform type, full-time versus part-time status, and perceived algorithmic control, were not examined. Future research should incorporate these variables to better identify the boundary conditions of organizational dehumanization in platform work. Finally, the present study examined a strain-based health impairment process within the JD-R framework. It did not include motivational mediators such as work engagement, meaningful work, or perceived autonomy. Future research should incorporate these motivational mechanisms to provide a fuller test of the JD-R dual-path model in gig work.

## 5 Conclusion and implications

This study examined whether organizational dehumanization is associated with Chinese gig workers’ flourishing and commitment to service quality through psychological distress, and whether family recognition of work moderates this process. The findings indicated that organizational dehumanization was positively associated with psychological distress, which in turn was negatively associated with both flourishing and commitment to service quality. Family recognition of work was associated with a weaker link between organizational dehumanization and psychological distress and with weaker indirect associations between organizational dehumanization and both outcomes through psychological distress. These findings suggest that family recognition of work may attenuate, but does not eliminate, the strain-based process associated with organizational dehumanization.

The study contributes to the literature in three ways. First, it extends organizational dehumanization research to platform-based gig work, where workers may lack stable organizational membership but remain subject to platform rules, algorithmic allocation, and performance evaluation. Second, it positions organizational dehumanization as a relational job demand within the JD-R framework and identifies psychological distress as a proximal strain mechanism linking dehumanizing platform experiences to worker outcomes. Third, it shows that family recognition of work may serve as a supplementary cross-domain resource, suggesting that resources relevant to gig workers’ strain processes may originate outside the workplace.

These findings also have practical implications. The primary intervention target should be platform practices that produce dehumanizing experiences. Consistent with critiques of algorithmic management that emphasize opacity, limited voice, and asymmetrical control in platform-like work systems (Kellogg et al., 2020), platforms can reduce instrumental treatment by improving the transparency of algorithmic management, explaining order allocation and rating rules more clearly, establishing humane appeal procedures to avoid purely automatic penalties, and increasing channels through which workers can communicate with human platform representatives. At the same time, community organizations, labor unions, or worker-support programs may help family members better understand the demands and social value of gig work. Such efforts may provide supplementary support for workers, but they should complement rather than replace platform-level responsibility for improving working conditions.

In summary, organizational dehumanization is associated with poorer wellbeing and weaker service-related commitment among Chinese gig workers partly through psychological distress. Family recognition of work appears to attenuate this strain-based process, but it should be understood as a supplementary buffer rather than a substitute for reducing dehumanizing conditions in platform work.

## Data Availability

The raw data supporting the conclusions of this article will be made available by the authors, without undue reservation.
